# Does the choice of neighbourhood supermarket access measure influence associations with individual-level fruit and vegetable consumption? A case study from Glasgow

**DOI:** 10.1186/1476-072X-11-29

**Published:** 2012-07-27

**Authors:** Lukar E Thornton, Jamie R Pearce, Laura Macdonald, Karen E Lamb, Anne Ellaway

**Affiliations:** 1 Centre for Physical Activity and Nutrition Research, School of Exercise and Nutrition Sciences, Deakin University, 221 Burwood Highway, Burwood, Victoria, 3125, Australia; 2 Centre for Research on Environment, Society and Health (CRESH), School of Geosciences, University of Edinburgh, Edinburgh, EH8 9XP, United Kingdom; 3 Medical Research Council Social and Public Health Sciences Unit, 4 Lilybank Gardens, Glasgow, G12 8RZ, Scotland, United Kingdom; 4 Clinical Epidemiology and Biostatistics Unit, Murdoch Childrens Research Institute, The Royal Children’s Hospital, Flemington Road, Parkville, Victoria, 3052, Australia

**Keywords:** Geographic information systems, Food environment, Dietary behaviours

## Abstract

**Background:**

Previous studies have provided mixed evidence with regards to associations between food store access and dietary outcomes. This study examines the most commonly applied measures of locational access to assess whether associations between supermarket access and fruit and vegetable consumption are affected by the choice of access measure and scale.

**Method:**

Supermarket location data from Glasgow, UK (n = 119), and fruit and vegetable intake data from the ‘Health and Well-Being’ Survey (n = 1041) were used to compare various measures of locational access. These exposure variables included proximity estimates (with different points-of-origin used to vary levels of aggregation) and density measures using three approaches (Euclidean and road network buffers and Kernel density estimation) at distances ranging from 0.4 km to 5 km. Further analysis was conducted to assess the impact of using smaller buffer sizes for individuals who did not own a car. Associations between these multiple access measures and fruit and vegetable consumption were estimated using linear regression models.

**Results:**

Levels of spatial aggregation did not impact on the proximity estimates. Counts of supermarkets within Euclidean buffers were associated with fruit and vegetable consumption at 1 km, 2 km and 3 km, and for our road network buffers at 2 km, 3 km, and 4 km. Kernel density estimates provided the strongest associations and were significant at a distance of 2 km, 3 km, 4 km and 5 km. Presence of a supermarket within 0.4 km of road network distance from where people lived was positively associated with fruit consumption amongst those without a car (coef. 0.657; s.e. 0.247; p0.008).

**Conclusions:**

The associations between locational access to supermarkets and individual-level dietary behaviour are sensitive to the method by which the food environment variable is captured. Care needs to be taken to ensure robust and conceptually appropriate measures of access are used and these should be grounded in a clear a priori reasoning.

## Background

Local residential environments are increasingly considered important factors in understanding health outcomes and behaviours [1,2]. For dietary behaviours, the local food environment provides the opportunity to purchase and consume both healthy and unhealthy foods [3]. For example, the presence of local supermarkets and greengrocers may facilitate the purchase of fresh fruits and vegetables at competitive prices whilst a greater number of fast food outlets increase the opportunities to purchase potentially unhealthy energy-dense items. To date, however, the evidence linking various aspects of the food environment to dietary behaviours and health outcomes remains mixed [4-6] with variations in access measures providing a possible explanation.

Urban destinations, including supermarkets, can be considered more geographically accessible when a lower travel cost in terms of distance, time, and/or financial resources is incurred [7,8]. Recent studies examining the relationship between the food environment and individual-level diet (and related health outcomes) have measured locational access using the following approaches: proximity to the nearest store [9-14]; presence/absence or number of food stores within an area (either administrative unit or a buffer around a specified location) [10,12,13,15]; and Kernel density estimation [16-20].

To summarise these measures, proximity estimates provide detail on travel distance to the nearest feature but provide no detail on the total number of facilities located nearby. Buffers can be generated to facilitate a count of features within a given distance. However, using a 2 kilometre (km) buffer as an example, one of the downsides to these measures is that having 4 facilities located within the first 0.4 km of the buffer is considered equal to having four facilities located between 1.5 km and 2 km away. Of course, smaller or even multiple sized buffers can be used but the binary nature of this approach (i.e. ≤2 km accessible; >2 km not accessible) may not always be appropriate. Kernel density estimates not only consider the number of features nearby, but a weighting function can also be applied so amenities which are closer are weighted more heavily than those located further away. Additionally, Kernel density estimates are created across a continuous surface so that density can be calculated from any location. However, one drawback to this approach is that the estimates are often calculated using Euclidean distance (straight-line) rather than road network distance meaning that potential travel barriers are not taken into account. Further details on these measures and their application in research on local environments and health have been reported elsewhere [7,21,22].

Additional variations within these different access measures can further complicate the interpretation of results. First, proximity and buffers can be estimated using either a straight-line or a network distance. Oliver and colleagues previously compared the use of straight-line and network buffers and demonstrated substantial differences in the association between exposures to land use characteristics and walking [23]. Second, there is limited theoretical grounding on which is the most appropriate distance to use for density estimates. Previously, some studies have undertaken analysis of buffers at multiple distances which has enabled the examination of distances that may be relevant for access via walking or driving [15,24,25]. Ideally, the relevant spatial scale of a buffer should be dictated by the choice of outcome, exposure, and the hypothesised causal pathway between the outcome and predictor [26-29]. Third, the point of origin from which proximity estimates and buffers are created may lead to aggregation error when a single point, usually a geometric centroid, is used to represent individuals spatially distributed within a boundary [30,31]. Aggregation of individual-level data can be related to the original data collection methods and may be unavoidable due to issues such as confidentiality [30,31]. The use of smaller geographic units and population-weighted centroids can help to reduce aggregation error because these estimates have a higher level of precision that more closely resembles the actual distance for each individual. However, their use will not totally eliminate this error [31,32]. The only way aggregation error can be avoided is by measuring from the micro-level of the individual (e.g. household location) [32]. Finally, it needs to be acknowledged that most measures to date only consider geographic access and few studies consider other factors that may influence an individual’s ability to travel to facilities; for example, public transport provisions and vehicle ownership [19,33].

The increasingly user-friendly nature of Geographic Information Systems (GIS) software and the ready availability of spatial data have seen the adoption of these geographic methods into epidemiological health research. GIS is recognised as a valuable tool for examining associations between characteristics of the built environment and health [4,5]. However, recent critiques of work in this field have suggested that GIS measures have often been developed without a clear theorisation of the processes they attempt to capture nor a firm understanding of the key principles of spatial analysis [4,6].

To date, the primary use of different exposure measures has been to determine if they explained disparities in food access across areas [19,34]. What remains largely unknown from current studies is what the effect these varied measures of access might have on the reported associations with diet-related outcomes [35-37]. The observation that different results might be obtained through using different spatial scales (or boundaries) is referred to as the Modifiable Areal Unit Problem (MAUP) [38]. We are not aware of prior studies that have comprehensively assessed how using different objective measures of access impacts on the associations with dietary outcome. If the appropriateness of access measures is not given due consideration prior to analysis then studies have an increased likelihood of conclusions with either Type-I errors (when a difference is said to exist but, in reality, does not) or Type-II errors (where a difference is stated not to exist, when in reality, it does).

This study undertakes a practical exploration of some of the commonly used measures of food store access and applies them to supermarkets in Glasgow, UK. Using individual-level data from the ‘Health and Well-Being’ Survey, associations between supermarket access and fruit and vegetable consumption were assessed using a variety of access measures. These include proximity estimates from different points-of-origin; Euclidean buffers, road network buffers and Kernel density estimates at different scales; and the consideration of an individual-level mobility indicator.

## Methods

### Study area and sample

Glasgow City is 68 square miles in size and in 2010 had a population of 592,820 [39] and contains 694 data zones (data zone: mean population 848, mean area 25.2 hectares). Data zones nest within local government boundaries and, where possible, were constructed to recognise physical boundaries (e.g. rivers) and identified communities [40]. Within Glasgow City in 2010, 96% of the data zones had a population of <200 per hectare and of the remaining 4%, the maximum people per hectare was 368.

This study capitalised on an existing dataset relating to Greater Glasgow, Scotland, the ‘Health and Well-Being’ (HWB) Survey conducted in 2002 by the Greater Glasgow Health Board (GGHB). The HWB sample was stratified proportionately by local authority and deprivation category (DEPCAT), with addresses selected randomly. Data were weighted to ensure that they were representative of the adult population in this area. Over two thirds (67%) of individuals contacted took part in the study which led to 1802 face-to-face interviews with adults [41]. Data were gathered on individuals’ socio-demographic characteristics, health and health behaviours [41] (and have previously been reported on [42]) and for this study only Glasgow City respondents were used (n = 1119 from 199 data zones).

### Fruit and vegetable consumption

Respondents to the HWB Survey were asked “How many portions of fruit do you eat each day?” and “How many portions of vegetables or salad (not counting potatoes) do you eat each day?” Examples of a portion size were provided for each question. Daily fruit and vegetable consumption was assessed separately in addition to total daily fruit and vegetable consumption combined; each as continuous variables.

### Exposure measures

For Glasgow City and the surrounding councils the addresses of 119 main chain supermarkets (Asda, the Co-op, Morrisons, Sainsbury’s, Somerfield, Tesco) were obtained by undertaking searches on the on-line yellow pages (http://www.yell.com/) (as at May 2010) which was supplemented and verified using the websites of the supermarket chains. Duplicates were identified and removed at this stage. Validation occurred using a combination of street view and local knowledge of supermarket localities. The size of supermarkets was not considered as this information was not available. As the buffers around individuals overlapped with the surrounding councils, the additional supermarket location data for surrounding councils was obtained to avoid the issue of ‘edge effects’. Spatial data for the study area, that is, the road network topology and data zone boundaries, were obtained from the UK Ordnance Survey [43] and the Scottish Executive [40] respectively. Geometric and population-weighted centroids (the point that minimises the total distance to all residents in an area [7]) were also obtained from the Scottish Executive (January 2010). GIS (ArcGIS v9.3) was used to geocode HWB respondents and food retailers by their unit postal codes. Respondents were geocoded to their postal code (using the geometric centroid) as we did not have their exact address. Unit postal codes typically contain around 15 address points and were therefore spatially proximate to the actual physical household address of the individual while still maintaining the confidentiality of the respondent’s household location. Each of the geocoded features were snapped to the nearest locality of the road network. From these data, a series of commonly used measures of food store access were created as detailed below.

#### Proximity to nearest supermarket

**Figure 1:**
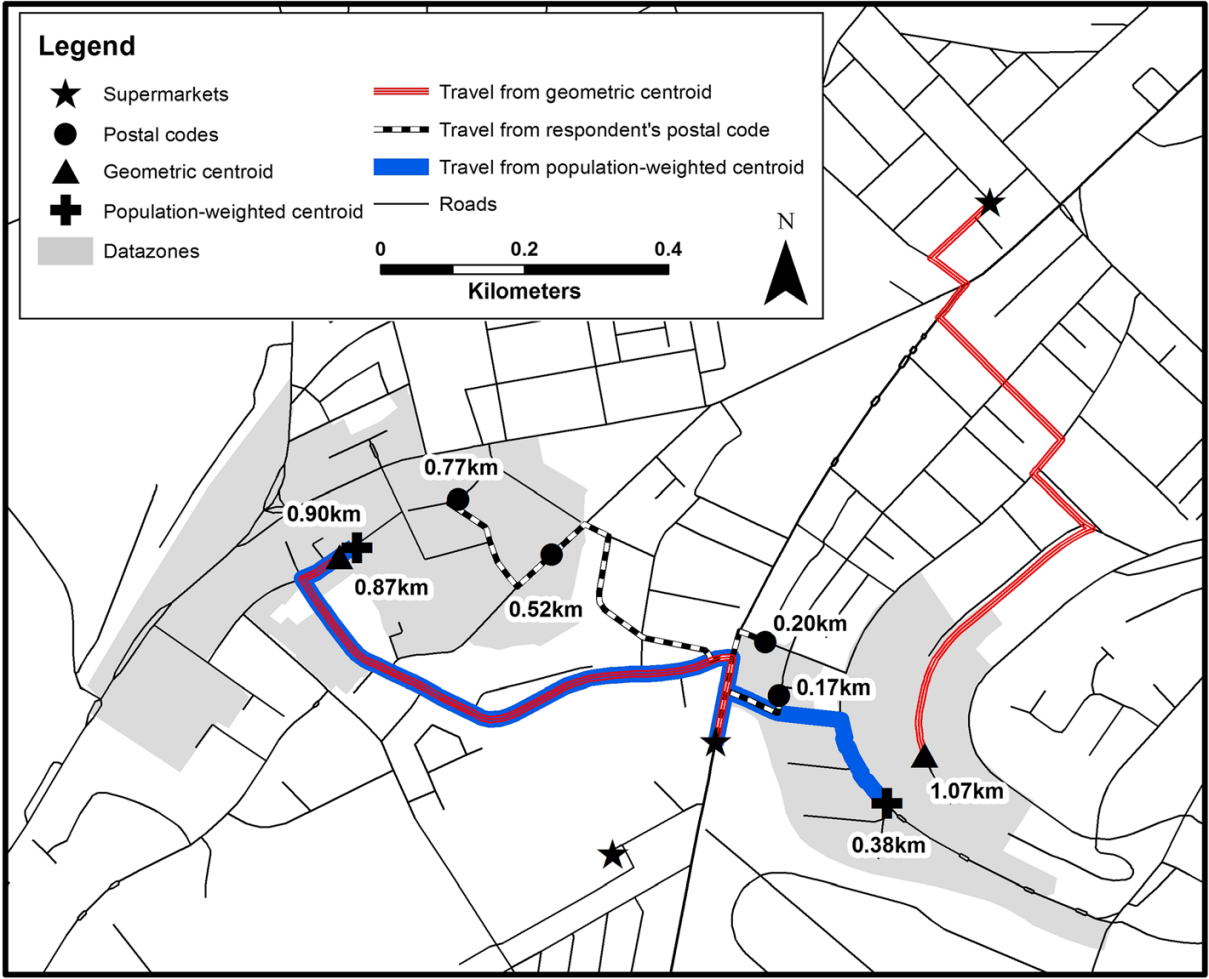
Road network distance proximity measures from geometric centroid, population-weighted centroid and postal codes to the nearest supermarket: An example of differences using two data zones.

Proximity was represented by calculating (using ArcGIS 9.3) the shortest distance along a road network from an origin to the nearest supermarket (Figure 1). Origin was measured from three points: 1) geometric centroid of the data zones; 2) population-weighted centroid of data zones; 3) unit postal code location. The use of a population-weighted centroid as opposed to a geometric centroid provided a greater specification of access for a higher number of individuals in that data zone. The final origin, unit postal codes, provided the least aggregated assessment of origin and consequently proximity.

#### Euclidean and road network buffers

Euclidean and road network distance buffers were created around the postal codes to assess the impact of exploring the presence/absence and count of supermarkets within the predefined distances. For the binary (presence/absence) measure, distances of 0.4 km, 1 km and 2 km were used as these represented buffer sizes used in prior research, and provided within unit variation of the proportion with and without a supermarket. This was not the case as the buffer size increased (for example, for the 3 km road distance buffer only 1% of the locations did not have access to a supermarket). Therefore, the 3 km, 4 km and 5 km buffers were not used in the analysis of presence/absence.

**Figure 2:**
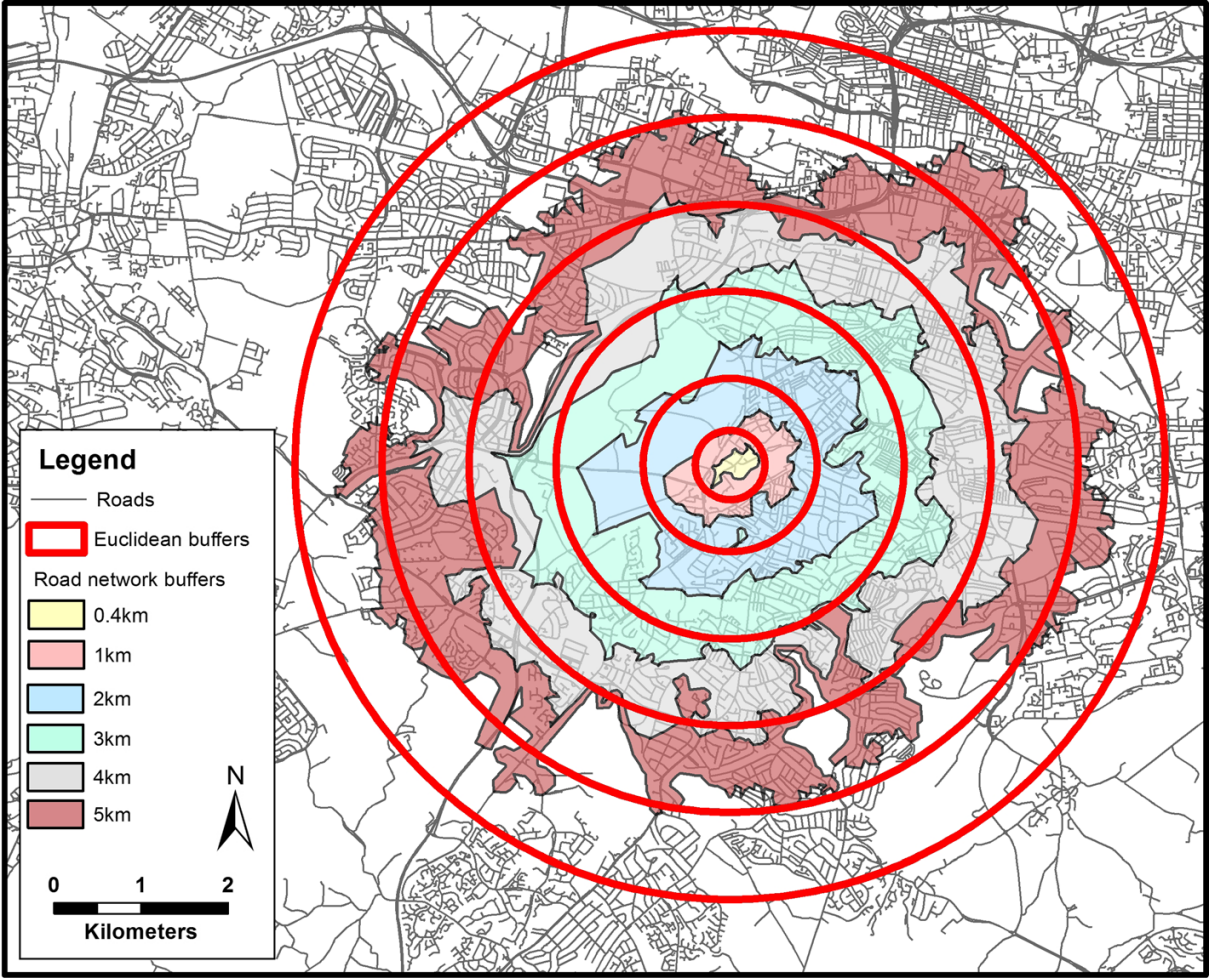
Differences in the geographic area covered by buffers of different scale and type.

When we examined the counts of supermarkets we used 0.4 km, 1 km, 2 km, 3 km, 4 km and 5 km Euclidean and road network buffers (Figure 2 demonstrates the different areas covered by these buffers). Count data differs from the assessment of a single supermarket as it represents the choice of options available to an individual in their neighbourhood. Using the count measures, we firstly assessed how associations with fruit and vegetable consumption differed when a straight-line measure of distance was compared to a distance that follows a road network path and, secondly, how associations differed when the spatial scale of the buffer size was varied.

#### Kernel density estimates

**Figure 3:**
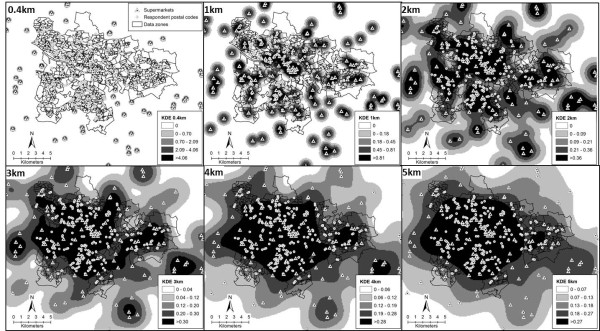
Kernel density estimations.

Kernel density estimates were created using the Kernel density tool in ArcGIS 9.3. The Kernel density function was used to calculate the density of supermarkets across a continuous surface for the extended study area (Glasgow City and surrounding councils) with the cell size set at 100 metres. This allowed a density estimate to be calculated from any point in the study area. This density value at any point represents the number of supermarkets nearby with those closer contributing a higher density value. For this analysis, Kernels of 0.4 km, 1 km, 2 km, 3 km, 4 km and 5 km were calculated and the density values at the unit postal code locations were assigned to the study participants according to area of residence (Figure 3). Kernel density estimates were generated using only Euclidean distances.

#### Individual-specific vs. uniform definition of scale

Individual factors are likely to influence accessibility by either restricting or promoting an individual’s mobility but are rarely considered. For example, not having access to a motor vehicle may act as a mobility barrier and restrict an individual to shopping within a nearer (walkable) distance to their home. In this instance, a restricted mobility may be detrimental to dietary behaviours if fresh produce is not accessible within the local area. In this study, vehicle access was measured by asking “do you, or any member of your household, own a car?” with response options being “yes” or “no”. Stratified analysis was used to determine access to supermarkets within 3 km and 5 km road network buffers for those with a car and 0.4 km and 1 km road network buffers for those without access to a car.

### Covariates

Respondents recorded their age, sex and highest level of educational qualification. Education was included as an indicator of socioeconomic position and was categorised to: 1) No formal qualifications; 2) Low (O-levels or equivalent); 3) Mid (A-levels or equivalent); 4) High (higher degree).

### Statistical analysis

Participants were excluded from analysis if they had missing data (n = 78) on one or more of the key variables, leaving a final sample of 1041. Multilevel linear regression models were analysed in Stata 11.2 [44]. Multilevel analysis was used to account for the clustering of individuals within data zones. Models were adjusted for age, sex and education.

## Results

### Sample characteristics

The average age of participants in this study was 50 years old (s.d. 20.2) (Table 1). The majority were female (61%) and lived in households that did not own a car (61%). A third of the sample reported having no formal educational qualifications and an additional 31% had only low educational qualifications. On average, participants consumed less than two portions of fruit and only two portions of vegetables per day.

**Table 1:** Sample characteristics

	**Mean**	**(s.d.)**
Age	50.5	(20.2)
Sex	n.	%
Female	639	61.4
Male	402	38.6
Educational qualifications		
No formal qualifications	359	34.5
Low	322	30.9
Mid	236	22.7
High	124	11.9
Vehicle ownership		
Yes	408	39.2
No	633	60.8
	Mean	(s.d.)
Fruit portions consumed (per day)	1.7	(1.6)
Vegetable portions consumed (per day)	2.0	(1.5)
Fruit and vegetables combined (per day)	3.7	(2.7)

### Supermarket access

#### Proximity

The mean distance to the nearest supermarket was ~1.2 km and minimal differences were observed between the three different points-of-origin (geometric centroid: mean 1.18 km, s.d. 0.59; population-weighted centroid: mean 1.17 km, s.d. 0.58; postal code: mean 1.16 km, s.d. 0.62) (Table 2).

**Table 2:** Measures of supermarket access

	**Mean (s.d.)**	**Median (IQR)**	**Range**
Proximity via road network from:			
Geometric centroid	1.18 (0.59)	1.06 (0.74–1.57)	0.02–3.78
Population-weighted centroid	1.17 (0.58)	1.06 (0.75–1.56)	0.18–3.25
Postal code	1.16 (0.62)	1.07 (0.70–1.56)	0.02–3.26
Percentage of individuals with a supermarket within:*			
0.4 km Euclidean buffer	23.5%		
1 km Euclidean buffer	72.7%		
2 km Euclidean buffer	100%		
0.4 km road network buffer	8.5%		
1 km road network buffer	42.8%		
2 km road network buffer	87.6%		
Count of a supermarket within:*			
0.4 km Euclidean buffer	0.29 (0.60)	0 (0–0)	0–5
1 km Euclidean buffer	1.42 (1.45)	1 (0–2)	0–9
2 km Euclidean buffer	5.31 (3.26)	5 (3–7)	1–19
3 km Euclidean buffer	11.53 (5.47)	9 (8–15)	3–26
4 km Euclidean buffer	20.36 (7.74)	18 (14–28)	8–35
5 km Euclidean buffer	29.70 (8.96)	31 (22–37)	12–45
0.4 km road network buffer	0.10 (0.36)	0 (0–0)	0–4
1 km road network buffer	0.70 (1.04)	0 (0–1)	0–7
2 km road network buffer	2.82 (2.82)	2 (1–4)	0–16
3 km road network buffer	6.51 (4.25)	6 (4–8)	0–22
4 km road network buffer	11.79 (6.21)	10 (7–16)	2–28
5 km road network buffer	18.81 (7.98)	17 (12–27)	4–36
Kernel density estimates			
0.4 km	0.57 (1.58)	0 (0–0)	0–12.75
1 km	0.48 (0.64)	0.27 (0–0.72)	0–5.00
2 km	0.43 (0.34)	0.38 (0.23–0.52)	0–2.14
3 km	0.43 (0.25)	0.35 (0.28–0.53)	0.09–1.51
4 km	0.42 (0.20)	0.38 (0.28–0.49)	0.12–1.10
5 km	0.41 (0.17)	0.37 (0.27–0.53)	0.14–0.84

*buffers and kernel density estimates based on individual’s postal code locations.

#### Buffers

Less than a quarter of the sample had a supermarket present within a 0.4 km Euclidean buffer but this increased to 73% at a distance of 1 km and 100% at 2 km (Table 2). As our road network buffers are smaller than the Euclidean buffers, these percentages were lower when network buffers were used for 0.4 km (9%), 1 km (43%), and 2 km (88%). For both Euclidean and road network distances, a greater variation in the counts of supermarkets were observed at a distance of 2 km and beyond. The count of supermarkets within 3 km, 4 km and 5 km road network buffers closely resembles the counts within Euclidean buffers of 2 km, 3 km, and 4 km, respectively.

#### Kernel density estimates

Values representing the Kernel density estimates are presented in Table 2. Maps displaying the Kernel density estimates for the Glasgow City area are displayed in Figure 3. Darker shades represent a higher density and density values are assigned to each individual based on their unit postal code location.

### Associations between supermarket access and fruit and vegetable intake

The next stage in the analysis was to examine the association between each of the supermarket access measures and the individual-level fruit and vegetable consumption.

#### Proximity to nearest supermarket

Levels of aggregation as a result of varying the point-of-origin for proximity estimates did not alter findings for the fruit and vegetable consumption outcomes with no statistically significant association detected (Table 3).

**Table 3:** Multilevel regression models examining association between fruit and vegetable consumption and varying measures of supermarket access

	**Fruit**			**Vegetable**			**Fruit and vegetables combined**		
	**coef.**	**s.e.**	**p**	**coef.**	**s.e.**	**p**	**coef.**	**s.e.**	**p**
Proximity via road network from:									
Geometric centroid	0.036	(0.110)	0.740	0.119	(0.117)	0.308	0.142	(0.205)	0.488
Population-weighted centroid	−0.026	(0.111)	0.814	0.076	(0.118)	0.520	0.039	(0.206)	0.851
Postal code	−0.069	(0.102)	0.503	0.051	(0.109)	0.639	−0.036	(0.190)	0.851
Presence of a supermarket within:									
0.4 km Euclidean buffer	−0.031	(0.144)	0.831	−0.117	(0.150)	0.437	−0.122	(0.264)	0.646
1 km Euclidean buffer	0.034	(0.137)	0.805	−0. 093	(0.142)	0.514	−0.056	(0.250)	0.824
0.4 km road network buffer	0.351	(0.201)	0.081	0.124	(0.206)	0.548	0.469	(0.359)	0.192
1 km road network buffer	0.067	(0.123)	0.587	0.083	(0.127)	0.516	0.184	(0.224)	0.410
2 km road network buffer	0.341	(0.189)	0.071	0.086	(0.200)	0.668	0.417	(0.350)	0.234
Count of a supermarket within:									
0.4 km Euclidean buffer	0.004	(0.099)	0.969	−0.078	(0.102)	0.444	−0.056	(0.179)	0.755
1 km Euclidean buffer	0.127	(0.040)	0.001	0.125	(0.042)	0.003	0.253	(0.073)	0.001
2 km Euclidean buffer	0.067	(0.018)	<0.001	0.068	(0.019)	<0.001	0.136	(0.032)	<0.001
3 km Euclidean buffer	0.023	(0.011)	0.034	0.022	(0.012)	0.063	0.047	(0.020)	0.022
4 km Euclidean buffer	0.010	(0.008)	0.190	0.002	(0.009)	0.840	0.014	(0.015)	0.347
5 km Euclidean buffer	0.008	(0.007)	0.284	−0.001	(0.007)	0.898	0.008	(0.013)	0.547
0.4 km road network buffer	0.139	(0.155)	0.369	0.042	(0.158)	0.790	0.181	(0.276)	0.512
1 km road network buffer	0.070	(0.057)	0.223	0.024	(0.059)	0.687	0.108	(0.104)	0.295
2 km road network buffer	0.104	(0.022)	<0.001	0.105	(0.024)	<0.001	0.213	(0.040)	<0.001
3 km road network buffer	0.054	(0.014)	<0.001	0.051	(0.015)	<0.001	0.107	(0.026)	<0.001
4 km road network buffer	0.023	(0.009)	0.017	0.017	(0.010)	0.100	0.042	(0.018)	0.019
5 km road network buffer	0.009	(0.008)	0.268	0.002	(0.008)	0.835	0.013	(0.015)	0.391
Kernel density estimates									
0.4 km Euclidean distance	0.021	(0.038)	0.587	−0.035	(0.040)	0.376	−0.009	(0.070)	0.896
1 km Euclidean distance	0.135	(0.094)	0.153	0.075	(0.099)	0.445	0.228	(0.173)	0.187
2 km Euclidean distance	0.627	(0.166)	<0.001	0.585	(0.176)	0.001	1.238	(0.303)	<0.001
3 km Euclidean distance	0.888	(0.233)	<0.001	0.902	(0.250)	<0.001	1.828	(0.430)	<0.001
4 km Euclidean distance	0.947	(0.303)	0.002	0.906	(0.327)	0.006	1.915	(0.563)	0.001
5 km Euclidean distance	0.899	(0.373)	0.016	0.699	(0.402)	0.082	1.687	(0.698)	0.016

Models adjusted for age, sex and education.

#### Supermarkets within buffers

The examination of the presence/absence of supermarkets as a binary indicator did not result in any statistically significant associations with fruit and vegetable consumption when examined within the Euclidean buffer distances of 0.4 km or 1 km and the road network buffers of 0.4 km, 1 km or 2 km (Table 3).

Using Euclidean distance buffers, the consumption of fruit was positively associated with the count of supermarkets within 1 km, 2 km and 3 km whilst vegetable consumption increased with a higher count of supermarkets within 1 km and 2 km (Table 3). The strongest association observed for the Euclidean buffers was for 1 km buffers for the fruits and vegetables combined measure (coef. 0.253; s.e. 0.073; p0.001) which was also statistically significant for the 2 km and 3 km buffers. For road network buffers, positive associations were found within 2 km, 3 km and 4 km for fruit, 2 km and 3 km for vegetables, and 2 km, 3 km and 4 km for fruits and vegetables combined. No significant associations were observed for 0.4 km, 4 km and 5 km Euclidean buffers or for 0.4 km, 1 km, and 5 km road network buffers which may reflect an absence of a sufficient gradient in exposure at these distances.

It was reported earlier that the counts of supermarkets within some Euclidean buffers were similar to those of road network buffers of a greater size. It is interesting to note that the magnitude of associations observed between counts of supermarkets and our outcomes were very similar in road network buffers that were 1 km larger than the Euclidean buffers. For example, for fruit consumption a coefficient of 0.127 (s.e. 0.040; p0.001) was recorded for the number of supermarkets within a 1 km Euclidean buffer whilst a coefficient of 0.104 (s.e. 0.022; p < 0.001) was observed for the 2 km road network buffer (Table 3). This pattern is repeated for the 2 km Euclidean buffer (coef. 0.067; s.e. 0.018; p < 0.001) and the 3 km road network buffer (coef. 0.054; s.e. 0.014; p < 0.001), as well as for the 3 km Euclidean buffer (coef. 0.023; s.e. 0.011; p0.034) and the 4 km road network buffer (0.023; s.e. 0.009; p0.017). Similar outcomes resulted for vegetable consumption and for fruits and vegetables combined.

#### Kernel density estimates

Significant associations between a higher density and greater consumption of fruit and fruits and vegetables combined were observed for distances between 2 km and 5 km (Table 3). For vegetables, statistically significant positive associations were only identified between 2 km and 4 km.

#### Individual specific vs. uniform definition

When only households without a car were assessed, having a supermarket within 0.4 km was associated with increased daily consumption of fruit portions per day (Table 4). In the full sample (both those with and without a car) this association did not reach statistical significance (Table 3). Amongst those with a car, the magnitude of associations and level of significance observed was similar to that amongst the full sample with a positive association found for 3 km road network buffers and no significant associations identified for 5 km road network buffers (Table 4).

**Table 4:** Multilevel regression models examining association between fruit and vegetable consumption and both the presence and count of supermarkets stratified by household vehicle ownership

	**Fruit**			**Vegetable**			**Fruit and vegetables combined**		
	**coef.**	**s.e.**	**p**	**coef.**	**s.e.**	**p**	**coef.**	**s.e.**	**p**
Households without a car (n. 633):									
Presence of supermarkets within 0.4 km road network buffer	0.657	(0.247)	0.008	0.113	(0.235)	0.631	0.777	(0.414)	0.060
Count of supermarkets within 0.4 km road network buffer	0.283	(0.184)	0.125	0.005	(0.172)	0.977	0.294	(0.305)	0.335
Presence of supermarkets within 1 km road network buffer	0.005	(0.162)	0.973	0.067	(0.151)	0.659	0.078	(0.269)	0.772
Count of supermarkets within 1 km road network buffer	0.077	(0.072)	0.280	−0.008	(0.067)	0.909	0.072	(0.119)	0.541
Households with a car (n. 408):									
Count of supermarkets within 3 km road network buffer	0.055	(0.023)	0.015	0.059	(0.024)	0.014	0.112	(0.041)	0.006
Count of supermarkets within 5 km road network buffer	0.009	(0.013)	0.465	−0.002	(0.014)	0.888	0.007	(0.024)	0.756

Models adjusted for age, sex and education.

## Discussion

In this Glasgow study, we found that the association between locational access to supermarkets and individual-level fruit and vegetable consumption was highly sensitive to the food environment measure that was selected. The findings suggest that, for a number of the access measures we created, greater access to supermarkets was associated with higher fruit and vegetable consumption. However, this was not the case for all measures; for example, these results suggest that no association is apparent when we assessed counts of supermarkets within a 5 km road network buffer around an individual’s unit postal code location. This finding draws attention to the potential risk of committing a Type-II error.

Whilst measuring from a unit postal code provided a less aggregated and, arguably, more precise estimate for proximity measures than the measures from the geometric or population-weighted centroids of the larger spatial units (data zones), the proximity estimates did not vary greatly across the whole sample by point-of-origin and were not associated with our three outcomes. Hewko *et al*. have previously noted that aggregation error is a greater concern when examining more densely populated features because proximity estimates are more sensitive in this instance [31]. Whilst supermarkets were reasonably densely populated in our study area, the analysis may have been more likely to detect an association if other more prominent food store types were examined (e.g. takeaway outlets) or a larger spatial unit was used rather than a data zone. However, given their size, using specific household addresses over postal codes would have only made minimal differences to these findings.

When the presence or absence of a feature is explored, the count data is dichotomised to examine whether any store is present within a set distance (e.g. is there a supermarket within 1 km). Again no significant association was observed using this approach amongst the full sample. However, when count data were investigated, some significant positive associations were detected, indicating a greater choice may be more important than access to a single store. Previously, greater choice in the form of different fast food chains has been linked to more frequent fast food use [13] suggesting that dietary behaviours are influenced by having access to a wider selection of options.

The strongest associations observed were when Kernel density estimates were used for the exposure measure. Chaix *et al*. previously posited that more often the use of a boundary (in this instance a buffer) implies a binary definition of access and that the use of a smooth transition between what is and is not accessible would more often reflect a truer representation of access [37]. The adoption of Kernel density estimation enabled the application of a smoothing process by weighting areas more heavily when they were proximal to other stores. This weighting diminishes when the number of stores nearby is reduced and/or the distance to other stores is increased. Once these estimates are created, individuals are plotted to this map and assigned a density estimate based on their location. Whilst recent examples of studies using Kernel methods in food environment research exist [16-20], it remains a relatively underutilised technique compared to standard proximity or buffer approaches.

Another interesting finding to emerge from our analysis was the similarity between 1 km, 2 km, and 3 km Euclidean buffers with 2 km, 3 km, and 4 km road network buffers, respectively. This suggests such measures may be comparable. Sparks *et al*. previously reported similar associations between Euclidian and road network buffers and concluded that disparate measures of food access can often be compared [34]. Consequently, they suggested that aggregated and Euclidean distance measurements offer the same outcomes as more sophisticated and potentially more resource intensive approaches (i.e. less aggregated data and road network measurements). However, contrasting findings are reported in a study using both Euclidean and network buffers to explore the role of land use on walking behaviours, with stronger associations found for network buffers [23], highlighting the need for clear conceptualisation of exposure measures prior to analysis.

The choice of distance for buffers varies considerably across studies assessing associations with the built environment [7,21,22]. However, the use of buffer distances that are too small can result in the lack of an adequate exposure gradient meaning the detection of an effect is unlikely [45]. Further, using distances that are too large often overestimate the exposure by capturing features that individuals are unlikely to interact with and again may reduce the heterogeneity of the exposure measure. Our study demonstrates that the scale of the exposure measure can have a considerable bearing on the interpretation of the existence or otherwise of a relationship. Inconsistencies in scales are mainly driven by a lack of data that can be used to inform researchers as to what distance should be explored. It has previously been argued that understanding “true” environmental differences requires the identification of “true” environments [46]. In this instance, defining a “true” environment would require us to know where people are being exposed to and buying food. Therefore, for an accurate assessment of the role of environmental influences on dietary behaviours, and for an improved conceptualisation of appropriate scales, it is essential that studies move from place-based to people-based measures of exposure [47].

It is also important that researchers begin to account for the wide variety of ways that individuals interact with their environment. Cummins noted that what constituted local and appropriate food access differs between individuals [48,49]. In this instance, it may be because some individuals will travel further to food stores that meet their needs (e.g. product variety and quality, specific ethnic stores, cheaper prices) [48,50]. More generally, socioeconomic factors are also likely to strongly influence an individual’s mobility and thus their ability to access particular food stores. For example, a low income can restrict motor vehicle ownership, potentially reducing an individual’s access to a wider variety of food stores [51,52]. To date, most investigations on local residential food environments and dietary behaviours are limited by the assumption that all stores within the local area are equally accessible to all residents irrespective of potential mobility barriers. Chaix *et al*. has called on future studies to consider the use of an individual-specific rather than uniform definition of neighbourhood scale [37]. Such analysis allows different scales to be applied based on individual characteristics. In our latter analysis, the exposure measure is further strengthened by considering an indicator of individual mobility through their vehicle ownership and use. When supermarkets within a walkable distance (0.4 km) were explored, an association was found with fruit consumption amongst those without a vehicle whereas when this was investigated amongst the full sample no relationship was detected. This suggests that considering personal mobility factors can strengthen our understanding of the links between the environment and health behaviours. Bader *et al*. previously explored the concept of “travel burden” whereby factors such as vehicle ownership, crime and public transit access are assessed to determine how these factors influence spatial access to healthy food.[19] Conducted in New York City, their study found that adjustment for vehicle ownership and crime tended to increase the observed disparities between neighbourhood race and income and supermarket access. Whilst their study did not examine links to health behaviours, a prior US study found stronger associations between local healthy food resources and insulin resistance amongst those who did not own an automobile [33].

This study was strengthened by the comprehensive assessment of multiple access measures, and more importantly, how these influence associations with dietary outcomes. Whilst some prior studies have compared how different access measure effect exposure estimates, they have not explored a range of measures and buffer distances as comprehensive as that undertaken in this present study nor have they assessed the impact on dietary outcomes. It is important that the use of outcome data is acknowledged as it provides some indicative data on comparability with other food environment studies that have used varying measures of access.

The limitations of this study must be acknowledged. First, with regards to the food environment only a single source of fruits and vegetables (supermarkets) was examined. Further, this study did not have within-store data on these supermarkets to help inform the quantity, quality and price of the fruits and vegetables sold which are potentially important factors in determining purchasing and consumption behaviours [53]. Additional factors at the area-level that may affect mobility (e.g. public transit option, crime, safety) were not considered nor were individual data related to perceptions of these factors. Our buffer estimates were all created based on distance metrics whereas additional data on speed limits may have allowed a more sophisticated approach that also included estimates of travel time. This is important to consider as it may be that time is more important than distance when considering how people interact with food stores or indeed there may be other factors such as individual’s preference for a particular area or store type that dictates where they shop. The cross-sectional nature of the data and the time lag between the individual survey data and the supermarket data may limit the applicability of the reported associations. However, first we reiterate that our primary aim was to demonstrate variations in associations based on different exposure measures rather than to establish causality between supermarket access and dietary outcomes. Second, with regards to the time lag, prior research demonstrates little change in the number of national ‘multiple-owned’ supermarkets in Glasgow between the years 1997 (n = 75) and 2007 (n = 78)[54] and therefore the static nature of chain-brand supermarket locations would likely mean this is unlikely to significantly influence the results. Finally, the data analysed is restricted to a single urban area in one country and findings may firstly, not be applicable in rural areas and, secondly, would require confirmation in other urban contexts elsewhere.

## Conclusions

The results of this study provided a working example of how our interpretation of associations between food environments and diet-related outcomes can differ depending upon the measurement of access and the scale employed. This serves to highlight the importance of a strong, a priori, conceptualisation of the exposure-outcome relationship when deciding on an appropriate access measure and to ensure that results are correctly interpreted and reported by investigators.

## Competing interests

The authors declare they have no competing interests.

## Author contributions

LT drove the conceptualisation of the study design, undertook the analysis, and wrote the first draft of this paper. JP, LM, KL and AE contributed to the study design and redrafting of the paper. Each author has read and approved the final version of this manuscript.
